# A Nanoparticle-based Sensor Platform for Cell Tracking and Status/Function Assessment

**DOI:** 10.1038/srep14768

**Published:** 2015-10-06

**Authors:** David Yeo, Christian Wiraja, Yon Jin Chuah, Yu Gao, Chenjie Xu

**Affiliations:** 1 School of Chemical & Biomedical Engineering, Nanyang Technological University, Singapore; 2 NTU-Northwestern Institute of Nanomedicine, Nanyang Technological University, Singapore

## Abstract

Nanoparticles are increasingly popular choices for labeling and tracking cells in biomedical applications such as cell therapy. However, all current types of nanoparticles fail to provide real-time, noninvasive monitoring of cell status and functions while often generating false positive signals. Herein, a nanosensor platform to track the real-time expression of specific biomarkers that correlate with cell status and functions is reported. Nanosensors are synthesized by encapsulating various sensor molecules within biodegradable polymeric nanoparticles. Upon intracellular entry, nanosensors reside within the cell cytoplasm, serving as a depot to continuously release sensor molecules for up to 30 days. In the absence of the target biomarkers, the released sensor molecules remain ‘Off’. When the biomarker(s) is expressed, a detectable signal is generated (On). As a proof-of-concept, three nanosensor formulations were synthesized to monitor cell viability, secretion of nitric oxide, and β-actin mRNA expression.

Cell tracking enables real-time visualization of biodistribution, migration and functional attributes of cells such as survival and differentiation^1^,^2^,^3^. The biodistribution and migration of cells has been well-studied through the development of passive contrast agents such as fluorescent proteins and magnetic nanoparticles (NPs)^4^,^5^. However, cell status and other functional attributes of implanted cells are not well understood due to inadequate cell labeling tools. Genomic modification with reporter genes is currently the only option to meet this need. For example, Lgr5^+^ intestinal stem cells can be distinguished from differentiated non-Lgr5 expressing lineages by a green fluorescent protein reporter controlled by the Lgr5 promoter region and a *β*-galactosidase-LacZ reporter (blue) to identify implanted cells. While Lgr5^+^ intestinal stem cells express both signals (green & blue), its non-stem cell daughter cells only express the blue signal. This system can further track reconstitution of the intestinal lining from a single stem cell^6^. Undoubtedly, reporter gene systems facilitate non-invasive and longitudinal tracking of cell behavior, and are indispensable to the progress of cell biology^1^,^7^,^8^. However, they are limited by low transfection efficiency in primary cells and stem cells, high experimental resource requirements, and concerns over risks resulting from random cell mutagenesis^9^,^10^.

Herein, we report an alternative approach that utilizes nanotechnology for cell tracking and status/function assessment without the above concerns. Specifically, a versatile nanoparticle platform (i.e. nanosensors) is developed to efficiently label a wide range of cell types with minimal changes to phenotype^11^. The nanosensors are prepared by encapsulating sensor molecules within biodegradable polymeric NPs. Thereafter, nanosensors are decorated with moieties to facilitate cell endocytosis. Although specific targeting molecules can be chosen, cationic poly-L-lysine was utilized in this study for efficient endocytosis *in vitro*. Free nanosensor particles were then separated from labeled cells. Upon intracellular entry, they reside within the cell cytoplasm, serving as a depot to continuously release sensor molecules. These sensors comprise organic fluorophore derivatives (e.g. Calcein Acetomethoxy (CAM)) and hydrophilic oligonucleotide probes designed to detect specific molecular sequences. In the absence of the biomarkers, the released sensor molecules remain ‘Off’. When the biomarker(s) is expressed, a detectable signal is generated (On). (Fig. 1).

## Results

**Figure 2:**
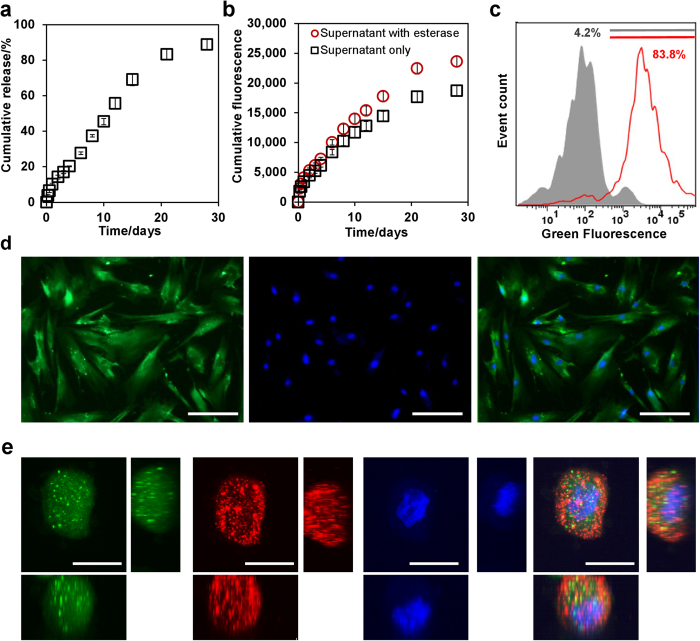
Viability nanosensors for labeling mesenchymal stem cells (MSCs). (**A**) Cumulative release of CAM from nanosensors over 28 days in PBS at 37 °C. (**B**) Fluorescence signal of supernatants in A before and after the esterase treatment. (**C**) Flow cytometry analysis of MSCs before (grey) and after (red) the labeling with 3 mg/ml viability nanosensor. (**D**) Nanosensor labeled MSCs stained with Hoechst 33342 (from left to right: nanosensors, nuclei, merged image). Scale bars are 100 μm. (**E**) Confocal images of nanosensor labeled MSCs stained with DiI and Hoechst 33342 (from left to right: nanosensors, plasma membrane, nuclei, merged image); Z-projection (main), YZ-plane (right), XZ-plane (below). Scale bars are 20 μm. Values are mean ± SD, N = 4.

To examine the feasibility of this idea, nanosensors designed to assess cell viability (i.e. viability nanosensors) were first synthesized by encapsulating CAM within poly(lactic-co-glycolic acid) (PLGA) NPs in the size range of 500 nm–1 μm (Supplementary Fig. S1a,b). When placed in phosphate buffered saline (PBS), a 10% release was observed during day 1 and 90% of encapsulated CAM molecules was subsequently released over 28 days (Fig. 2a). This was quantified using an absorption-concentration calibration curve of free CAM (Supplementary Fig. S1c). CAM is known to be weakly fluorescent, but converts to strongly fluorescent calcein in the presence of esterases. When the aqueous supernatant was treated with esterases, we observed a ~20% increase of fluorescent intensity (Fig. 2b, Supplementary Fig. S1d). Compared to freshly constituted CAM (over ~120% enhancement with the esterase treatment, Supplementary Fig. S1e,f), the released CAM sensors exhibited reduced signal enhancement. This reduced functionality was in all likelihood, a result of spontaneous CAM hydrolysis in aqueous solution^12^.

Viability nanosensors were next used to label and track mesenchymal stem cells (MSCs). Cell labeling efficiency is influenced by parameters including size, surface charge, and concentration of NPs. Previously, we have shown that 400 nm–2 μm particles have prolonged cellular retention in MSCs and a positive surface charge facilitates higher labeling efficiency^13^. Thus, nanosensors throughout this study were kept within 500 nm–1 μm in size and modified with cationic poly-L-lysine (PLL). As shown in Supplementary Fig. S2a,b, MSCs labeled with PLL-modified nanosensors showed higher fluorescence compared with cells labeled with unmodified nanosensors under all concentrations, which confirmed the importance of cationic coating during cell labeling. The effect of labeling concentration was next examined, in which a 33-fold increase in fluorescence intensity was observed when nanosensor concentration increased from 0.01 to 5 mg/ml (Supplementary Fig. S2b). This concentration-dependent signal increase was further confirmed through flow cytometry, where labeling efficiency was higher in 3 mg/ml (Fig. 2c) nanosensor concentration (83.8%) compared with that (73.2%) in 0.3 mg/ml (Supplementary Fig. S2c). More impressively, this 10-fold difference in labeling concentration contributed to a ~60-fold difference in the median fluorescence intensity (Supplementary Fig. S2c), suggesting that a higher nanosensor internalization rate occurred with higher labeling concentrations. Henceforth, nanosensor labeling concentration was set at 3 mg/ml to maximize the labeling efficiency and intracellular nanosensor concentration.

Following labeling, nanosensors entered the cell cytoplasm and slowly released CAM molecules that reacted with intracellular esterases to express a fluorescence signal, as an indirect measure of cell viability (Fig. 2d). This labeling was observed to persist during routine subculture in which enzymatic disassociation and re-plating did not diminish signal intensity from nanosensor labeled MSCs (Supplementary Fig. S2d). Nanosensor localization was further examined with confocal imaging, in which DiIC_18_(3) (DiI, red) and Hoechst 33342 (blue) counter-stained the plasma membrane and nucleus respectively. As shown in Fig. 2e, nanosensor viability signal was well distributed throughout the entire cytoplasm. Certain areas expressing higher green signal were expected to be endosome/lysosome containing nanosensors, since they overlaid with red signal labeled plasma membrane.

**Figure 3:**
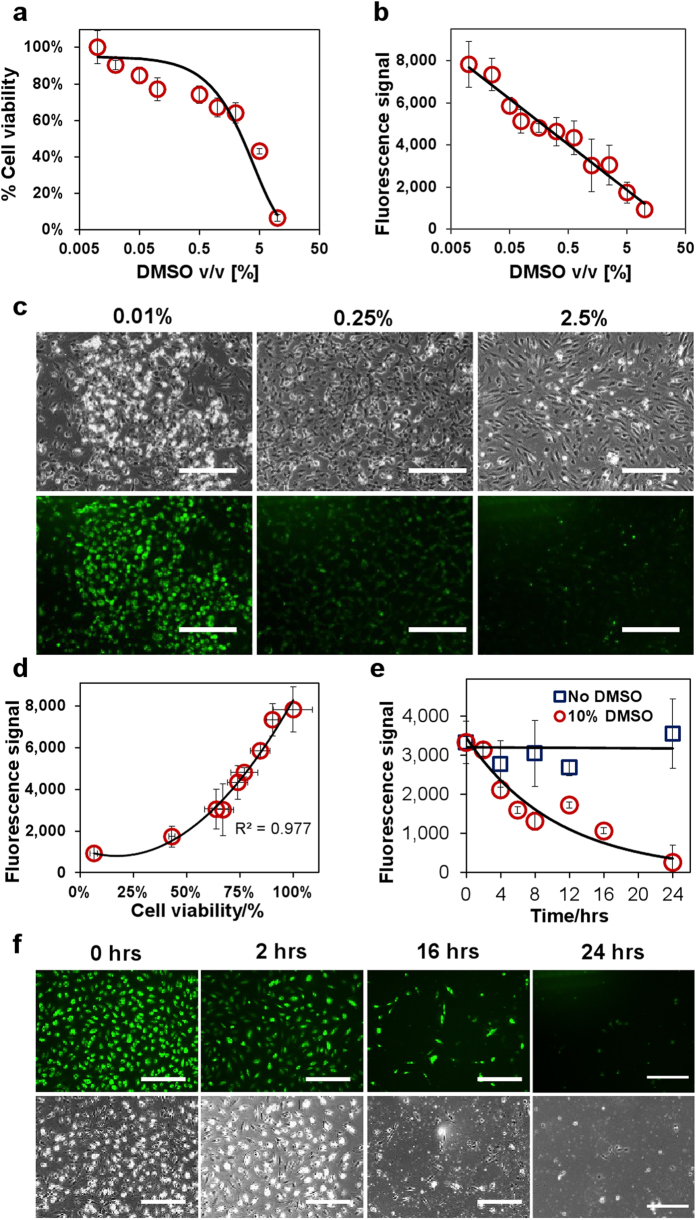
Non-invasive monitoring of cell viability using nanosensors. (**A**) Concentration-dependent cytotoxicity of DMSO (0.01–10% v/v) determined by CAM assay. (**B**) Fluorescence intensity of nanosensor labeled cells in response to the increasing DMSO concentration level (0.01–10% v/v). (**C**) Phase contrast and fluorescence images of nanosensor labeled cells 24 hours post-treatment with 0.01%, 0.25% & 2.5% (v/v) DMSO. (**D**) Correlation between fluorescence intensity of nanosensor labeled cells and cell viability. (**E**) Real-time monitoring cell viability with nanosensors in response to the 10% DMSO exposure. (**F**) Representative fluorescence and phase contrast images of (**E**). Values are mean ± SD, N ≥ 3. Scale bars represent 100 μm.

Viability nanosensors were next assessed for their ability to monitor cell survival. Although 10% dimethyl sulfoxide (DMSO) is commonly used in cryopreservation, solutions containing 2–10% DMSO are known to be detrimental to cell health^14^. CAM labeling is a reliable assay to gauge cell viability since the generated fluorescence signal is directly proportional to the quantity of endogenous esterases – an indicator of cell health^12^. Free CAM assay readings revealed that 10% DMSO concentration levels treated for a 24 hour period caused 94 ± 8.0% of cells to become non-viable (Fig. 3a). Cell counting further reveals a concentration-dependent loss of cell viability, with 26 ± 2.1% and 77 ± 8.1% decrease in cell numbers for 0.25% and 5% (v/v) DMSO treatment respectively (Supplementary Fig. S3a). Similarly, nanosensor labeled cells treated with increasing DMSO (0.01–10% v/v) concentration levels led to a 95 ± 4.6% decrease in fluorescence signal (Fig. 3b). Representative images (0.01, 0.25 & 2.5% v/v) reflected this progressive loss of fluorescence signal with increasing DMSO concentrations (Fig. 3c). To validate nanosensor performance, acquired signal (Fig. 3b) was compared with results obtained from the CAM assay (Fig. 3a) at matching DMSO concentration levels (Fig. 3d). A good correlation between signal intensity and cell viability was observed (R^2^ = 0.977).

In addition to the end-point analysis, nanosensors were further used to monitor cell viability in real-time. As a proof-of-concept, viability nanosensor-labeled cells were treated with solutions containing 10% (v/v) DMSO for a 24 hour period (Fig. 3e, Supplementary Fig. S3b). After 6 hours incubation, the fluorescence signal decreased to 49 ± 5.9% of the original intensity, detected through microplate readings. Further incubation (until 24 hours) exacerbated the signal decline to 10 ± 5.9% of its original levels. Representative images (Fig. 3f) reflect this gradual decline in nanosensor signal corresponding with the loss of adherent cells. In comparison, control groups maintained a steady fluorescence signal throughout the same time period (Supplementary Fig. S3b). Thus, we demonstrate how nanosensors can non-invasively ascertain cell viability.

**Figure 4:**
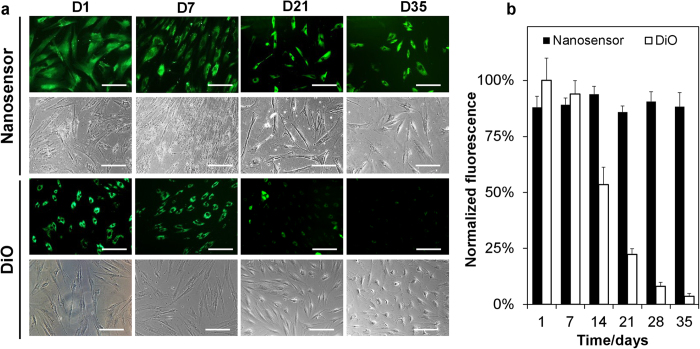
Longitudinal tracking of nanosensor labeled mesenchymal stem cells (MSCs). (**A**) Representative fluorescence and phase contrast images of nanosensor and DiO labeled MSCs during a culture period of 35 days post labeling. (**B**) Normalized fluorescence intensity from nanosensor and DiO labeled MSCs in **A**. Scale bars represent 100 μm. (N = 6, >200 cells analyzed).

Longitudinal studies are typically required to monitor and understand the behavior of transplanted cells, since certain events such as cell differentiation occur over a period ranging from weeks to even months^15^. To explore the potential of viability nanosensors for longitudinal cell tracking, nanosensor-labeled MSCs were monitored for a 2 month period, while being compared with cells labeled with DiOC_18_(3) (DiO), a commercial contrast agent for cellular imaging (Fig. 4)^16^. Unlike nanosensors, DiO is a lipophilic carbocyanine dye that labels the cell plasma membrane. At day 1, there were similar percentage of fluorescent cells in the nanosensors and DiO labeled groups (~92%) (Supplementary S4a,b). Both DiO and nanosensor labeled cells expressed similar fluorescence intensity per cell at this stage (Fig. 4a,b). After 14 days, there was only 70% DiO-labeled cells with detectable fluorescence signal while 85% nanosensor-labeled cells were fluorescent (Supplementary Fig. S4b). More impressively, the average signal of the fluorescent cells decreased approximately 50% for DiO-labeled cells during the 14 day period, yet the signal from nanosensor-labeled cells negligibly decreased (Fig. 4b). Subsequently, average fluorescence intensity from DiO labeled cells continued to decrease weekly (Fig. 4b). In contrast, nanosensor labeled MSCs maintained signal levels at a stable level without significant intensity decrease. Nanosensor labeled MSCs continued to be imaged for a total of 8 weeks (56 days), with nanosensor labeled cells expressing a stable signal throughout (Supplementary Fig. S4c). This dramatic difference between nanosensor and DiO labeled cells demonstrates the advantages of its sustained release mechanism, enabling prolonged cellular retention of imaging contrast.

The influence of contrast agents on the host cells is a critical concern, since changes to cell phenotype/properties can diminish therapeutic efficacy^17^,^18^. Crucially, the impairment of MSC functional properties (e.g. migration, differentiation etc) has previously been observed following labeling with various particles^19^,^20^. Therefore, we assessed the proliferation, ‘stemness’ and differentiation properties of nanosensor labeled MSCs to examine the effect of nanosensor labeling on MSC functional properties.

Through counting adherent cells post labeling, we observed that viability nanosensors did not significantly compromise proliferation rates compared to unmodified MSCs (Supplementary Fig. S5a). Colony forming unit-fibroblast (CFU-F) assay revealed that nanosensor labeled MSCs generated fibroblast colonies to a similar extent as unlabeled MSCs (Supplementary Fig. S5b)^19^. MSCs with and without nanosensor modification were next subjected to the following differentiation assays: AdipoRed (adipogenesis), Alcian Blue and collagen type II (chondrogenesis), and von Kossa (osteogenesis) assays (Supplementary Fig. S5c). No visible compromise to MSC differentiation ability could be observed. Furthermore, lipid vacuoles evident in both nanosensor and unmodified MSCs suggested that MSCs retained adipogenic potential after labeling (Supplementary Fig. S5d). To further validate MSC differentiation potential, gene expression analysis was performed using real-time polymerase chain reaction (PCR). Accordingly, both labeled and unlabeled MSCs expressed a similar level of *Alkaline Phosphatase* and *Collagen I* and *Osteonectin* (osteogenic markers), *Collagen II and Sox9 (*chondrogenic markers) (Supplementary Fig. S5e,f), ascertaining the minimal influence of nanosensors on MSC phenotype. Unexpectedly, a higher expression of *Aggrecan* (a late-stage chondrogenic marker)^20^ was observed in nanosensor-labeled MSCs, suggesting the positive effect of NP labeling on chondrogenesis (Supplementary Fig. S5f). The biological significance of this result however, is beyond the scope of this report. This series of tests reveal the minimal influence of nanosensor labeling on MSC phenotype, and allay concerns over bio-imaging agent safety.

The nanosensor platform can be further extended to monitor other endogenous functional molecules. For example, nitric oxide (NO) plays a critical role as a secondary biochemical messenger in numerous physiological angiogenic, cardiovascular, neurological and immune processes^21^. Successful monitoring of NO generation within live cells can serve as an early surrogate biomarker for therapeutic cell functionality. NO nanosensors were synthesized by encapsulating 4-amino-5-methylamino-2′,7′-difluorescein diacetate (DAF-FM-DA) within PLGA NPs. In the presence of intracellular esterases, released DAF-FM-DA sensor is deacetylated into 4-amino-5-methylamino-2′,7′-difluorescein (DAF-FM) which binds NO and becomes strongly fluorescent^22^.

**Figure 5:**
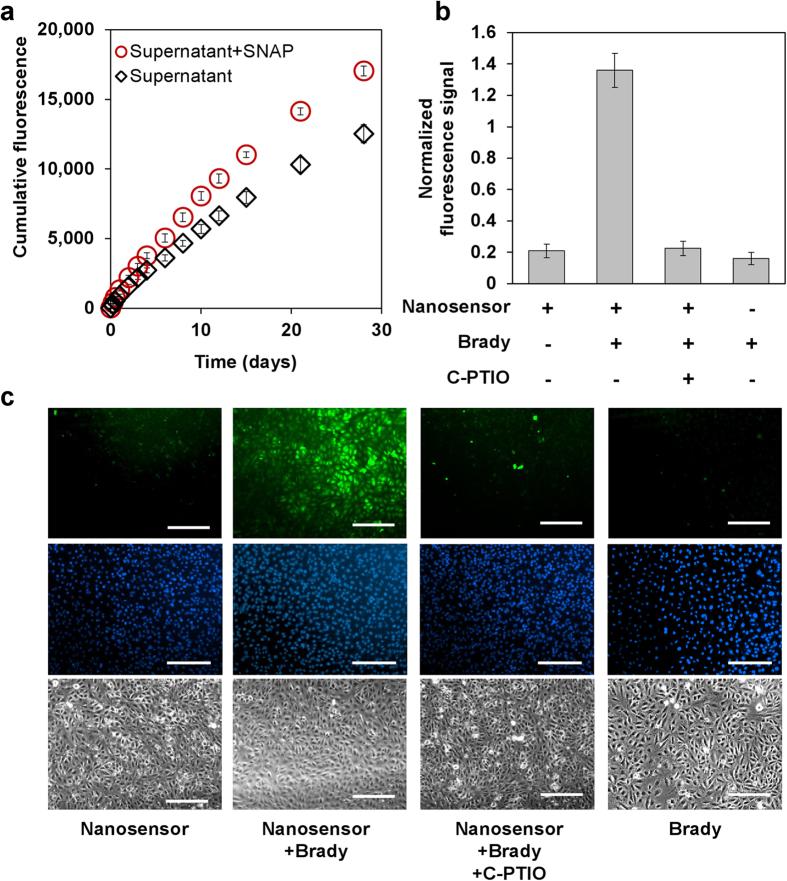
Nanosensors for Nitric Oxide (NO) detection. (**A**) Functional assessment of DAF-FM DA molecules eluted from nanosensors using SNAP. (**B**) Fluorescence from NO nanosensor-labeled HUVECs in response to bradykinin (Brady) and NO inhibitor, carboxy-PTIO (C-PTIO) treatment normalized to total cell numbers. Green signals were from nanosensors while blue signals were from nuclei staining. (**C**) Representative images of fluorescence and phase contrast images of nanosensor-labeled HUVECs following Brady and/or C-PTIO treatment. Values are mean ± SD, N ≥ 3. Scale bars represent 100 μm.

During NO nanosensor incubation within aqueous solution, a steady release of DAF-FM-DA was observed for at least 28 days (Supplementary Fig. S6a). Similar to CAM released from viability nanosensors (Fig. 2b), free DAF-FM-DA deacetylated in aqueous solution^23^. The addition of the NO donor S-Nitroso-N-acetyl-DL-penicillamine (SNAP) resulted in a ~40% signal intensity increase, demonstrating that functionality was preserved in released DAF-FM-DA (Fig. 5a). Thereafter, MSCs were labeled with NO nanosensors to evaluate their performance in live cells. Since MSCs did not generate NO at detectable levels^24^, they were treated with SNAP which served as an exogenous NO donor. As seen in Supplementary Fig. S6b, NO nanosensors and SNAP individually did not trigger fluorescence from cells, but in combination fluorescence was detected. Having ascertained their responsiveness to NO, the NO nanosensors were next applied to detect endogenously produced NO. Endothelial cells such as human umbilical vein endothelial cells (HUVECs) respond to bradykinin peptides by increasing calcium signaling that in turn triggers NO generation through NO synthase (NOS)^25^. On the other hand, NOS activity and NO species are inhibited by the NO scavenger carboxy-PTIO (C-PITO), generating NO_2_ as a by-product^26^. As shown in Fig. 5b,c fluorescence signal (normalized by total cell numbers) of nanosensor modified HUVECs remained at basal level without any treatment. The addition of Bradykinin (Brady) resulted in a 7-fold increase in fluorescence. In turn, the addition of the NO inhibitor carboxy-PTIO nullified the signal, keeping it at basal levels. Groups treated with a single addition of either nanosensor or Brady did not express signal levels higher than the baseline.

In addition to the hydrophobic sensor molecules used above, the nanosensor platform is compatible with delivering hydrophilic oligonucleotide molecule sensors that function as gene expression nanosensors. Oligonucleotides are highly attractive for molecular recognition due to their ease and cost-effectiveness in synthesis as well as high specificity^27^. Various delivery methods have been used to transport oligonucleotides beyond the plasma membrane to enable interaction and subsequent non-invasive detection of intracellular messenger RNA (mRNA). Current delivery methods rely on bolus (one-time) oligonucleotide delivery either by transiently generating pores on the plasma membrane (i.e. streptolysin, SLO) or using a secondary carrier to permit intracellular entry (e.g. lipofectamine® etc.). A significant limitation is the need for repeated loading steps (every ≤3 days)^28^ of sensor molecules, that may cause cytotoxicity^29^.

Nanosensors to detect mRNA were synthesized by encapsulating oligonucleotide molecular beacon sensors (MBs) within the PLGA particles through the double emulsion method (MB nanosensors). As a case study, oligonucleotide MB sensors (Table 1) complementary with β-actin mRNA, a gene commonly used as a housekeeping control^30^,^31^, was chosen as the nanosensor payload. Upon hybridization with its target mRNA (i.e. β-actin mRNA), MBs separate their 5′ and 3′ ends. This distances quencher and fluorophore moieties, restoring the pre-quenched fluorescence signal. Sensor specificity was demonstrated by a significant reduction in signal expression when the perfect target was replaced by a single base-pair mismatched oligonucleotide sequence (Supplementary Fig. S7a). Both perfect and single base-pair mismatch target sequences are documented in Table 1. Additionally, a MB sensor with a recognition sequence that does not hybridize with any known mRNA species in human MSCs (Scrambled) was used to develop a control MB-nanosensor.

**Figure 6:**
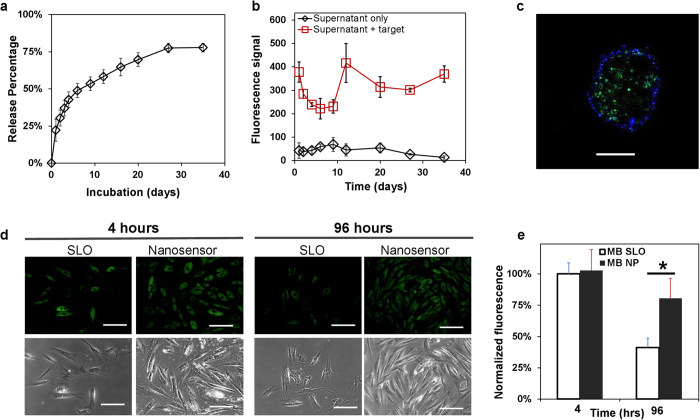
β-actin mRNA nanosensors for imaging β-actin mRNA in MSCs. (**A**) Sustained release of MBs from β-actin mRNA nanosensors over a 35 day period. (**B**) β-actin mRNA binding assay for supernatants in (**A**). (**C**) Confocal image of β-actin mRNA nanosensor labeled MSCs 4 hours post labeling. Blue is from stained cytoplasm membrane and green represents nanosensor signal. Scale bar represents 20 μm. (**D**) Fluorescence and phase contrast images of MSCs at 4 hours and 96 hours after being labeled with SLO-MBs or nanosensors. Scale bars represent 100 μm. (**E**) Normalized fluorescence intensity per cell in D *p < 0.05, n ≥ 150 cells. Values are mean ± SD, N ≥ 3.

To examine the reproducibility of nanosensor fabrication, 5 batches of nanosensor formulations containing β-actin & Scrambled MBs were independently fabricated. As a measure of process quality, encapsulation efficiency was quantified using UV-VIS to detect unencapsulated MBs. This was found to have little variation between batches with an average of 82.0 ± 0.06% encapsulation efficiency (Supplementary Fig. S7b). Sustained release of MBs from nanosensors was examined in aqueous solution by quantifying the total eluted quantity. Over a 35 day period, a cumulative release of ~75% of encapsulated MBs was observed (Fig. 6a). The addition of a complementary oligonucleotide—matching the β-actin mRNA sequence into the supernatant resulted in >8-fold increase in fluorescence signal (Fig. 6b), demonstrating the preservation of MB functionality following particle encapsulation and subsequent release.

The nanosensors enabled successful MB delivery, resulting in an intracellular fluorescence signal that signified β-actin mRNA detection (Fig. 6c). As a control, MSCs were labeled with free MBs through SLO transfection (Supplementary Fig. S7c). Immediately following labeling, MBs delivered through both nanosensor and SLO methods showed similar fluorescence intensity (Fig. 6d,e), signifying successful detection of β-actin mRNA with released MBs. After a 4 day period, whereas the fluorescence signal from nanosensor labeled cells did not significantly decrease, >50% loss of signal intensity was observed from the control SLO group. This demonstrated that nanosensors could deliver and release MBs to detect specific mRNA elements with great specificity. Furthermore, sustained MB release outperforms conventional bolus delivery methods (i.e. SLO) for longitudinal mRNA detection. High detection specificity is illustrated by evidence that Scrambled MB nanosensors (sequence found in Table 1) did not express any fluorescence signal (Supplementary Fig. S7d).

Utilizing ‘off’ Scrambled MB nanosensors present a unique opportunity to study sensor molecule (i.e. MBs) intracellular/extracellular distribution. These ‘off’ sensors do not emit signals due to the black hole quencher (BHQ) moiety which preserves reporter signal photo-stability. To quantify sensor molecule intracellular/extracellular distribution, enzymatic cell lysates (proteinase K) and cell supernatant are collected. The change in fluorescence signal measured before and after DNase I digestion is then compared to a standard curve obtained using known quantities of sensor molecules (Supplementary Fig. S7e). Similar to release kinetics within the aqueous solution, intracellular sensor molecule concentration was consistent throughout the experimental period suggesting that sensor molecules were released at a uniform intracellular rate. On the other hand, extracellular sensor concentration levels were ~6.8-fold lower than intracellular (Supplementary Fig. S7f).

## Discussion

Nanoparticle-based contrast agents are increasingly being used for cell imaging and tracking in fundamental, pre-clinical and clinical studies. However, none of them are able to monitor cell status/function continuously therefore cannot report the status of cells administered as therapeutics. Herein, we address this need by encapsulating sensor molecules within biodegradable polymeric NPs to create nanosensors. These nanosensors are engineered to enter the cell membrane, serving as intracellular reservoirs for sustained sensor molecule release to detect specific intracellular biomarkers revealing status/function in live cells.

The nanosensor platform is highly versatile with nanosensors synthesized using commercially available polymers (i.e. PLGA) and sensor molecules (e.g. CAM, DAF-FM DA, and MBs) through single or double emulsion methods. As a proof-of-concept, viability and NO nanosensors were synthesized by encapsulating hydrophobic CAM and DAF-FM DA sensor molecules respectively within PLGA by single emulsion, whereas double emulsion method was required for loading hydrophilic oligonucleotide β-actin MB sensors into PLGA particles. After PLL-modification, nanosensors efficiently labeled MSCs (Figs 2d, 4a, 6c & S2a, S4a,c, S6b, S7b), HUVECs (Figs 3b, 5c & S3b) and THP-1 monocytic cells (*data to be published*)—stem, primary and suspension cells respectively. Difficulties are commonly experienced during the transfection of these cells using common transfection agents like lipofectamine^32^,^33^,^34^, but we show that they can be easily labeled using nanosensors.

Given the advances made in drug delivery using biodegradable polymers, various nanosensor particle composition^35^ and processing parameters (e.g. particle coating^36^, emulsifier^37^, payload concentration^38^ etc.) can be engineered to achieve the ideal release kinetics for sensor payloads of different sizes and chemistries. Although not specifically demonstrated in this study, the release rate of encapsulated sensor molecules possibly influences nanosensor signal. In consideration of this, nanosensors were loaded with excess quantities of sensor molecules to saturate any available biomarkers. For example, nanosensors were loaded with calcein AM at 300-fold^39^ and DAF-DM DA 12-fold^22^ its normal usage concentrations for detecting viability and NO respectively. Intracellular availability of sensor molecules (using Scrambled MB nanosensors) was found to be highly consistent (9 days) with no substantial burst release (Supplementary Figure S7e,f). To mitigate against differential sensor molecule release at various experimental time-points, reference signals can be incorporated to normalize against signals generated from biomarkers of interest for accurate signal acquisition. Herein, Hoechst 33342 nuclear staining was utilized as a reference signal, in particular for Fig. 5b,c. However, it was only applied during end-point imaging, since it can interfere with live cell behavior. A better method to acquire reference signals could involve using nanosensors to detect expression levels of ‘housekeeping’ genes that are normally expressed with little fluctuation (e.g. glyceraldehyde-3-phosphate dehydrogenase).

Upon encapsulating sensor molecules within NPs, a significant concern was the potential loss of activity. To evaluate this, nanosensors were incubated in buffer solutions for a period of 30 days, and the supernatant was collected and analyzed for their ability to recognize targets of interest (Figs 2b, 5a, 6b). For example, CAM released from viability nanosensors (Fig. 2a) experienced a significant loss (5-fold) in signal enhancement following esterase treatment, in comparison to the fresh molecules. The loss of functionality is in all likelihood due to spontaneous hydrolysis of CAM and DAF-FM DA following release from NPs, having been reported for similar organic fluorophore derivatives^12^. On the other hand, released oligonucleotide MB sensors expressed a strong signal enhancement ratio. Following the combination with their perfect target, an almost 8-fold increase in fluorescence signal is obtained which suggests their greater stability makes them more suitable as sensor payloads.

The viability nanosensor successfully monitored changes in cell viability during treatment with solutions containing DMSO at different concentration levels (with fixed period) and different exposure periods (with fixed concentration levels) (Fig. 3). Exposure to DMSO causes molecules encapsulated within PLGA particles to become rapidly release^40^, which may boost intracellular CAM availability and overestimate cell viability. However, the good correlation (R^2^ = 0.977) between nanosensor signal and end-point free CAM suggests that DMSO usage as a cytotoxic agent does not significantly interfere with nanosensor performance. NO nanosensors successfully detected exogenous and endogenously generated NO species (Fig. 5, Supplementary S6). This signal was shown to be highly specific, since its signal was expressed only in the presence of an NO donor (SNAP) or stimuli for endogenous NO generation (Bradykinin). A known NO inhibitor, C-PTIO suppressed this signal, further demonstrating nanosensor specificity. High signal specificity was likewise observed for mRNA nanosensors. This was evident in a single base-pair sequence mismatch causing decreased signal expression levels (Supplementary Fig. S7a). Sequence specificity is underscored by the perfect target and the (single nucleotide) mismatched target having E values of 0.023 and 5.7 (BLAST®) respectively for *homo sapien* β-actin mRNA. Within live cells, control non-specific sequence MB loaded nanosensors generated negligible signals—suggesting that false positive signals negligibly contribute to β-actin nanosensor signal (Fig. 6, Supplementary S7).

Biopolymer degradation is a critical feature of the nanosensor since it enables sustained sensor molecule release directly within the cell cytoplasm. As discussed above, sensor molecules were gradually released over a period of at least 30 days. Sustained sensor release is a major factor for prolonged signal persistence (at least 2 months) observed in MSCs. Enhanced fluorophore photostability due to polymeric encapsulation^41^ is yet another advantage of the nanosensor platform. Remarkably, this lasts for an 8-fold longer period compared to DiO labeling (Fig. 4b). The original DiO signal was only maintained for 7 days, thereafter it decreased by approximately 2-fold every subsequent week (Fig. 4). This decrease results from MSC proliferation activity causing a consequential decrease in labeled plasma membrane area upon division into daughter cells as well as poor photo-stability. Nanosensor size (500–1,000 μm), is yet another factor promoting prolonged intracellular retention^4^,^13^,^42^. Thus, sustained release in nanosensors is advantageous for reducing the extent of signal dilution during cell labeling.

Unavoidably, *in vivo* nanosensor usage should be discussed, considering eventual therapeutic application^3^,^4^. Interaction of nanosensors with immune cells is a critical consideration, given their known interaction with PLGA particles^43^. Based on the current labeling strategy, free nanosensor particles are removed prior to usage. Although loss of cell viability risks nanosensor leakage, minimal quantities of nanosensor is likely to be exposed to immune cells following implantation. Given that cell internalized nanosensors experience similar mechanical and chemical microenvironments (cell cytoplasm) whether *in vitro* or *in vivo*, we anticipate that introducing nanosensor labeled cells into pre-clinical animal models would not alter its release properties, nor compromise nanosensor detection capabilities.

Sustained oligonucleotide MB release was highly beneficial, eliminating the need for successive rounds of oligonucleotide delivery—the current manner in which it is performed^28^ which decreases experimental interference as well as potential delivery agent cytotoxicity (e.g. SLO^29^). MB nanosensors are a highly promising direction since novel molecular targets can be detected by simply altering oligonucleotide recognition sequence. An immediate goal is to utilize MB-nanosensors for tracking stem cell differentiation in tissue engineering/regenerative medicine applications by detecting expression levels of highly-defined molecular events^28^. Nanosensor flexibility in the intracellular delivery of both hydrophilic and hydrophobic molecules suggests that other dyes, peptides, proteins are amenable as sensor payloads. This suggests that alternative detection strategies such as: SNAP-/CLIP-tag^8^ and RNA aptamers^44^ may be similarly applied for detecting other cellular status/function.

Finally, it deserves mention that the current version of nanosensors is limited to optical imaging. Biological tissues (e.g. mucosa, hepatocyte cells) and growth media contain many components (e.g. enzymes: flavins, NAD(P)H; extracellular matrix: collagen and elastin; phenol red), that emit autofluorescence which confound fluorescence signal specificity^45^. Further development of nanosensors is necessary using red-shifted fluorophores^46^, longer-lasting fluorophores (e.g. quantum dots^47^, nanodiamonds^48^) or non-optical sensor molecule alternatives. In conclusion, we have developed a non-integrative, easy-to-use, versatile nanotechnology platform for imaging live cell behavior. This advance simplifies and enhances cell tracking by enabling real-time acquisition of status/function.

## Methods

All materials except otherwise stated were purchased from Sigma-Aldrich. Dulbecco’s modified eagle medium (DMEM) high glucose with L-glutamine was purchased from Lonza. Fetal bovine serum (FBS), Trypsin-EDTA (0.05%), and penicillin-streptomycin (10,000 U/ml) and DNase/RNase-free distilled water and TRIzol® reagent were purchased from Invitrogen.

### Cell culture

Human mesenchymal stem cells (hMSCs) harvested from normal human bone marrow were purchased from Lonza which is pre-validated to be positive for: CD105, CD166, CD29 and CD44; negative for CD14, CD34 and CD45. These were cultured in hMSC growth media (PT-3001) supplemented with 10% fetal bovine serum (FBS, Sigma), 1% penicillin-streptomycin at 37 °C with 5% CO_2_. These were used between the passages 2–7. Differentiation experiments were performed between passages 3–5. Human umbilical vein endothelial cells (HUVECs) were purchased from Life Technologies (Singapore). HUVECs were seeded on collagen coated plates and cultured in EGM-2 media (Lonza, Singapore). Medium change was performed every 2–3 days and cells used were between passages 5–9.

### Nanosensor fabrication

Nanosensors were synthesized through single or double emulsion methods^4^. For viability and NO nanosensors, 250 μg CAM or 1 mg DAF-FM DA in 2 ml chloroform was mixed with 100 mg PLGA (50:50) at 4 °C. This mixture was added dropwise to 3% polyvinyl alcohol (PVA) aqueous solution and homogenized (Tissue Master 125, Omni International) for 60 s. The emulsion was placed in the chemical hood to evaporate chloroform for 3 hrs. Finally, nanosensors were collected through centrifugation (6,000 rpm) and washed 3× with double-distilled water before freezing-drying.

To generate β-actin mRNA nanosensors, 0.1 nmol MBs were first dissolved in 100 μl double-distilled water and homogenized into 500 μl chloroform containing 10 mg of 50:50 PLGA with 1% Span^®^ 80. This water-oil emulsion was further homogenized with 3% PVA to form a water-oil-water double emulsion. All other fabrication steps were similar to the single emulsion as above.

### Sensor release and functionality evaluation

To evaluate the release of sensor molecules (CAM, DAF-FM DA, or MBs), nanosensors were dispersed in PBS at 37 °C. The absorbance of the supernatants at 495 nm was obtained at different time points using a UV-2450 UV-Visible spectrophotometer (Shimadzu). Quantity of eluted sensor molecules was estimated by normalizing absorbance readings with standard curves generated from known quantities of sensor molecules. Percentage values are obtained by normalizing released quantities with the original loaded quantity.

To evaluate sensor functionality following particle encapsulation and release, fluorescence signal from the collected supernatant was measured at the indicated time point (Genios FL plate reader (TECAN)), before and after the addition of the sensor molecule target. In particular, the different sensor molecules and their specific targets were incubated for 30 minutes before signal acquisition. The following are the sensor molecules and their targets: (1) CAM sensors with 1 U/ml esterases, (2) DAF-FM DA sensors with 62.5 μM SNAP, which spontaneously decomposes to generate NO (0.88 μM NO/minute), (3) β-actin MBs with 0.5 μM β-actin target sequence.

### Scanning Electron Microscope

Lyophilized nanosensors were plasma-coated with gold for 180 s and then imaged on JSM-6700F field emission scanning electron microscope (FESEM, JEOL) (5 kV).

### Hydrodynamic diameter characterization

The hydrodynamic diameter of nanosensors was quantified with Zetasizer nano Z (Malvern). Briefly, 1 mg of nanosensors were dispersed in 1 ml double-distilled H_2_O and introduced to the zetasizer. At least three measurements were performed.

### Cell labeling

#### Calcein AM

 Cells at a density near confluence (80–95%) were labeled using a free Calcein AM (2 μM) solution. Following 30 minutes incubation cells were washed and rinsed with PBS and fresh medium added.

#### Nanosensor labeling

3 mg nanosensors were placed in 1 ml PLL solution (0.1% w/v) for 20–30 minutes. Then PLL coated nanosensors were separated through centrifugation and re-dispersed in 1 ml cell culture medium. Cells were incubated with nanosensor-containing medium overnight before washing 3× with PBS to remove unbound nanosensors.

#### Free MB labeling

Cells at a density near confluence (80–95%) were made permeable by incubating 6 U/ml SLO with 0.1 nM MBs for 10 minutes at 37 °C. To stop the permeabilization the cells were washed 3× with DMEM + 10% FBS, before allowing the cell membrane to reseal at 37 °C for 30 min.

#### Carbocyanine dye labeling

Lipophilic carbocyanine dye, DiO was used to label cell membrane. Briefly, 5 μl of DiO labeling solution was mixed per 1 ml of culture medium and added to adherent cells near confluence (80–95%). Alternatively, cells can be labeled as a cell suspension following trypsin dissociation. Following 20 minutes incubation time, cells were washed with PBS and fresh medium added.

### Quantifying extracellular/intracellular sensors

Confluent MSCs (24-well-plate wells) were labeled with nanosensor (loaded with scrambled Cy5.5 MBs) at 1.5 mg/ml particle concentration. 24 hr post-labeling, excess particles were rinsed with PBS. 400 μl fresh culture medium was then added for all groups for subsequent incubation. At designated time-points (day 1, 3, 5, 7, and 9), cell supernatant was collected (to retrieve extracellular sensors), and the cell pellet stored for subsequent enzymatic (proteinase K) cell digestion (to retrieve intracellular sensors). The protocol was based on the following^49^. Briefly, collected cell pellets were incubated overnight in 200 μl Proteinase K solution (50 μg/ml, Sigma) in 100 mM dibasic potassium phosphate buffer (pH 8) at 37 °C before heat-inactivation through incubation at 75 °C for 15 minutes and storage in 4 °C. To quantify MB concentration, 100 μl of supernatant or digested cells were treated with DNAse I (40 U/ml) for 20 minutes prior to detection using a fluorescence microplate reader (excitation/emission: 674/695 nm, optimal gain, 20 flashes/read). The RFU values obtained were then fitted into the calibration curve (generated by lysing MB molecules using DNAse I).

### Fluorescence imaging

Fluorescent images were acquired using a LX71 inverted fluorescent microscope (Olympus). The same camera settings (1,000 ms exposure time and 3× gain) were used to capture all fluorescent images, at 100× image magnification.

#### Image analysis

Fluorescence intensity of cells was quantified using the ImageJ software. Briefly, signal intensity of individual cells was acquired and averaged (≥150 cells) to obtain the mean ± standard deviation (SD) value at indicated time-points. 1 or 2-way ANOVA was carried out to calculate the *P*-value. A suitable post hoc test was chosen using the SigmaStat 3.5 software.

#### Population-averaged fluorescence detection

Emission of fluorescence signal from labeled cells was measured using a multi-plate reader using either 48- or 96 well plates. These are population average values obtained per well using a Genios FL plate reader (TECAN) on FITC channel (excitation 485 nm and emission at 515 nm). Immediately before reading, the fluid contents from each well are emptied to enable direct exposure to the excitation laser. Values are read based on the ‘optimal gain’ and normalized to negative (unlabeled cells).

### Confocal imaging

Sample preparation followed a protocol we published previously^42^. Briefly, nanosensor labeled cells were trypsinized and washed with 4 °C PBS once. Then cells were suspended at a density of 1 × 10^6^/mL in membrane dye working solution (5 μM Vybrant DiI or DiB, Life Technologies) for 5 minutes at 37 °C. After washing 3× with cell culture medium, cells were re-seeded on fibronectin-coated cover slips for 20 minutes. Then cells were fixed with fresh-prepared chilled 4% paraformaldehyde in PBS for 10 minutes on ice. Later, NucBlue® Live cell stain was used to stain the nucleus before mounting onto microscope slides for visualization with confocal microscope LSM710 (Zeiss).

### Flow cytometry

Approximately 1 million of nanosensor-labeled cells were washed in PBS, re-suspended in cold FBS until the flow cytometry analysis using the LSR Fortessa™ X-20 Flow cytometer (Becton Dickinson). Unlabeled cells and nanosensor particles were used as gating control samples to distinguish unbounded nanosensors and labeled cells.

### MSC differentiation assays

Differentiation was performed in accordance to the protocol from the manufacturers (Poietics^TM^ human mesenchymal stem cells).

#### Adipogenic differentiation

Fully confluent monolayers of MSCs were subjected to 3 cycles of induction/maintenance for approximately 3 days per cycle. Induction media contained the following supplements: h-insulin, dexamethasone, indomethacin, 3-isobutyl-l-methyl-xanthine (IBMX), whereas maintenance media contained h-insulin. Thereafter, MSCs were cultured for 7 more days in adipogenic maintenance media. Successful adiogenesis was noted by the observation of lipid vacuoles in the cells.

#### Chondrogenic differentiation

Complete chondrogenic medium contains dexamethasone, ascorbate, insulin-transferrin-sodium selenium supplements, proline, and 10 ng/ml Transforming Growth Factor β3 (TGF-β3). ≥2.5 × 10^6^ dissociated MSCs were washed with chondrogenic medium without TGF-β3 once before centrifuging to form a cell pellet. These were then incubated with complete chondrogenic medium within polypropylene centrifuge tubes, and maintained in the cell incubator with loosened tube caps. Every 2–3 days, the media was exchanged for fresh complete chondrogenic medium. Chondrogenic pellets were harvested for qPCR analysis and histology after 28 days.

#### Osteogenic differentiation

MSCs were plated at a density of 3.1 × 10^3^ cells/cm^2^ before osteogenic differentiation was induced. Osteogenic medium containing dexamethasone, ascorbate, β-glycerophosphate, was replaced every 3–4 days for a period of 21 days before qPCR analysis and alizarin red staining.

### Quantitative Polymerase Chain Reaction (qPCR) analysis

Total RNA was isolated using the RNeasy Mini Kit (QIAGEN) by following the manufacturer’s instructions. Prior to RNA isolation, chondrogenic pellets were first digested in DMEM containing 0.25% collagenase at 37 °C for 3 hours. Quantity was determined via NanoDrop (NanoDrop Technologies). RNA (200 ng) was reverse transcribed into cDNA products using an iScript DNA Synthesis Kit.

To ascertain the relative level of mRNA expression, a real-time PCR assay was performed (Applied Biosystems) using the SYBR Green PCR Master Mix Kit (Life Technologies). Primers specific to gene markers that represented the desired lineages were used to amplify the product cDNAs from reverse transcription. All primer sequences for qPCR can be found in Table 2.

#### Real-time PCR was performed using the following conditions

95 °C for 10 minutes, followed by 40-cycle amplification consisting of a denaturation step at 95 °C for 15 seconds, and an extension step at 60 °C for 1 minute. All data was normalized to GAPDH mRNA expression levels, and expressed as mRNA relative change using the 2^−∆∆CT^ method^50^ with reference to undifferentiated MSCs prior to the addition of respective differentiation stimuli.

### Histological staining

Osteogenic and chondrogenic specimens were fixed in 10% formalin overnight. Chondrogenic pellets were embedded in Tissue Freezing Medium (Leica Microsystems) and frozen at −20 °C. 8 μm sections of chondrogenic pellets were using with a Leica CM1900 cryostat (Leica Microsystems) and collected on Mezel-Glaser Superfrost Plus Slides (Thermo Scientific). Both the fixed specimens were washed with distilled water or 1X PBS before staining accordingly.

#### Alcian blue

To evaluate glycosaminoglycan expression using alcian blue staining, deparaffinized sections were immersed with 0.5% alcian blue in 0,1 M HCl. To evaluate the levels of collagen types II in different experimental groups, section slides were blocked with hydrogen peroxide before 20 min pepsin treatment.

#### Collagen II

Slides were also submerged in monoclonal antibodies of collagen types II of a 1:500 dilution factor for an hour, followed by incubation with biotinylated goat anti-mouse for 30 min. Streptavidin peroxidase was subsequently added for 45 min and 3, 30-diaminobenzidine were used as a chromogenic agent. Counterstaining was performed with Gill’s hematoxylin.

#### Alizarin red

To detect calcium deposits from osteogenesis, cells were immersed in Alizarin red solution for 5 min followed by gentle washing with distilled water until nonspecific staining was removed. All stained slides were dehydrated before placing on cover slips. Slides were then observed on a colour microscope and images acquired.

## Additional Information

**How to cite this article**: Yeo, D. *et al.* A Nanoparticle-based Sensor Platform for Cell Tracking and Status/Function Assessment. *Sci. Rep.* **5**, 14768; doi: 10.1038/srep14768 (2015).

## Supplementary Material

Supplementary Information

## Figures and Tables

**Figure 1:**
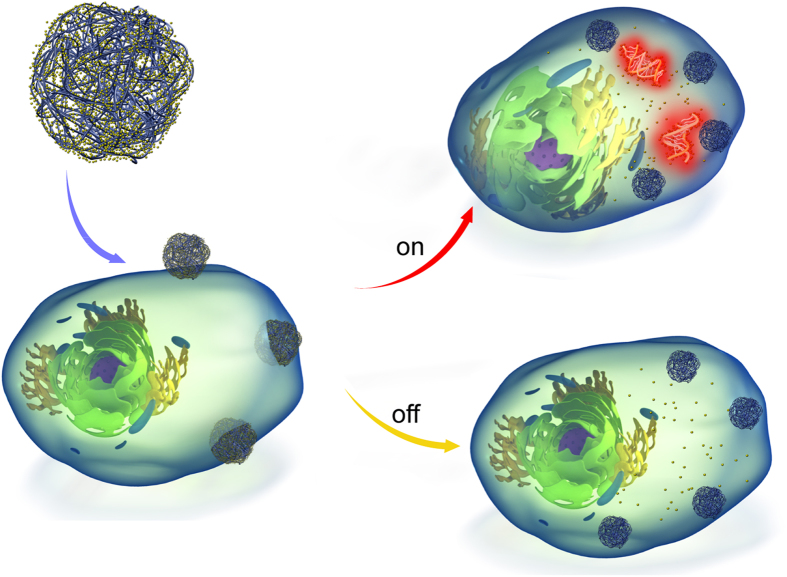
Schematic illustration of the nanosensor platform and intracellular implementation.

**Table 1:** The MB for β-actin and their complementary/mismatch target sequences were adopted from previous work^51^.

Oligonucleotides	Sequences (5′ to 3′)
Human β-actin MB	[Flc]-CCCGA-GCGGCGATATCATCATCCAT-TCGGG-[BHQ1]
Scrambled MB	[Flc/Cy5.5]-CCCGA-CGACAAGCGCACCGATATGAC-TCGGG-[BHQ1]
β-actin perfect match	ATGGATGATGATATCGCCGC
β-actin single mismatch	ATGG**T**TGATGATATCGCCGC

For comparison purposes, scrambled MB of a similar length with no known mRNA target (verified using BLAST) was used. *MB: molecular beacon, Flc: Fluorescein, BHQ: black hole quencher. The single mismatched nucleotide has been bolded and underlined.

**Table 2:** Primer sequences used for qPCR analysis, gene name and its direction (Forward/Reverse, F/R) are indicated below.

Oligonucleotides	Sequences
Sox9 (F)	AGTACCCGCACTTGCACAA
Sox9 (R)	CTCGTTCAGAAGTCTCCAGAGCTT
Aggrecan (F)	ACTTCCGCTGGTCAGATGGA
Aggrecan (R)	TCTCGTGCCAGATCATCACC
Col2 (F)	GGCAATAGCAGGTTCACGTACA
Col2 (R)	CGATAACAGTCTTGCCCCACTT
Osteonectin (F)	CGCCTGGGTCTCTTCACTAC
Osteonectin (R)	CTCACACTCCTCGCCCTATT
ALP (F)	ACCACCACGAGAGTGAACCA
ALP (R)	CGTTGTCTGAGTACCAGTCCC
Col1 (F)	CAGCCGCTTCACCTACAGC
Col1 (R)	TTTTGTATTCAATCACTGTCTTGCC
GapDH (F)	ATGGGGAAGGTGAAGGTCG
GapDH (R)	TAAAAGCAGCCCTGGTGACC
