# Supplementary material for: A Nanoparticle-based Sensor Platform for Cell Tracking and Status/Function Assessment
Source: Sci Rep. 2015 Oct 6;5:14768. doi: 10.1038/srep14768 (PMC4593999; doi:10.1038/srep14768)
Supplement: Supplementary Information [file srep14768-s1.pdf]

Supplementary Information

A Nanoparticle-based Sensor Platform for Cell Tracking and Status/Function Assessment

David Yeo[1], Christian Wiraja[1], Roger Chuah Yon Jin[1], Gao Yu[1], Chenjie Xu[1,2,★]

[1] School of Chemical & Biomedical Engineering, Nanyang Technological University, Singapore
[2] NTU-Northwestern Institute of Nanomedicine, Nanyang Technological University, Singapore
Correspondence and requests for materials should be addressed to C.J.X. (CJXu@ntu.edu.sg)

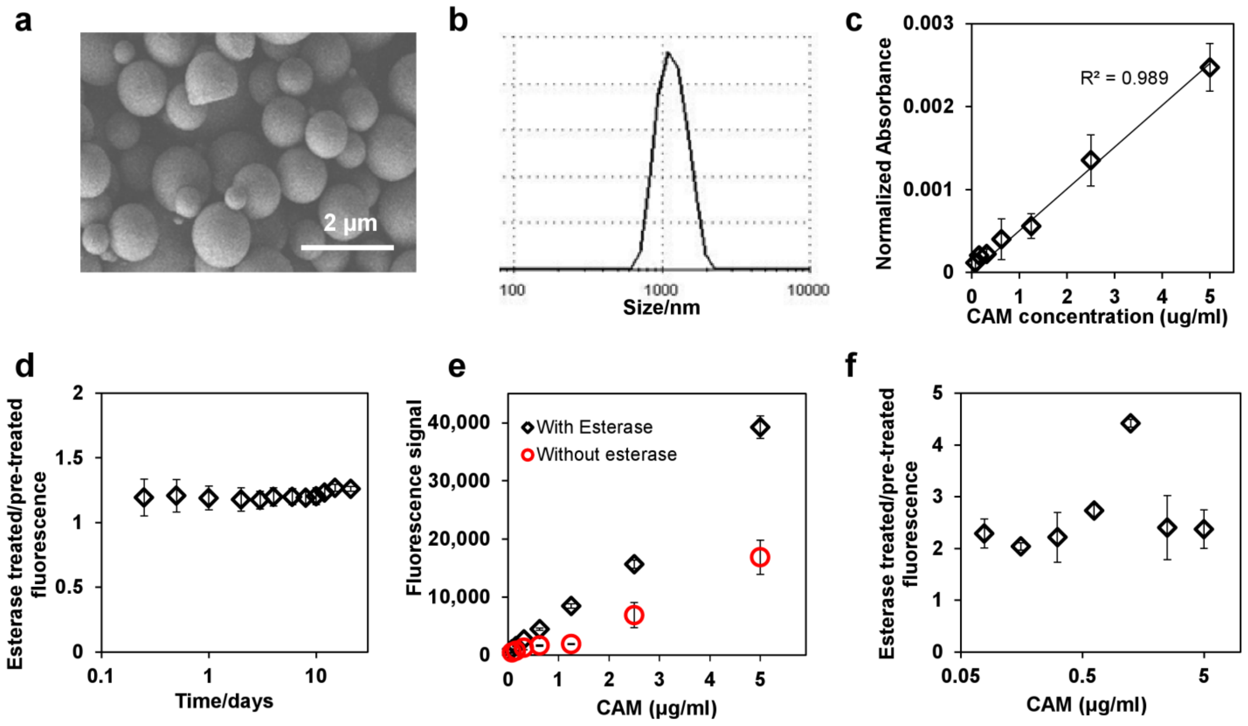

Supplementary Figure S1. Physical characterization of viability nanosensors. (**A**) Representative scanning electron microscope (SEM) image of nanosensor particles. (**B**) Hydrodynamic diameter of nanosensor measured through dynamic light scattering. (**C**) Calibration curve of UV absorbance at 495nm versus known calcein acetomethoxy (CAM) concentrations. (**D**) Ratio of fluorescence signal from esterase-treated supernatant versus untreated supernatant over a 28 day period. (**E**) Fluorescence signal (relative fluorescence units – RFU) from known quantities of CAM (0.078 – 5μg/ml) before and after the esterase treatment. (**F**) The ratio of fluorescence signal from esterase-treated CAM versus untreated CAM in **E**. All values are mean ± SD, N≥3.

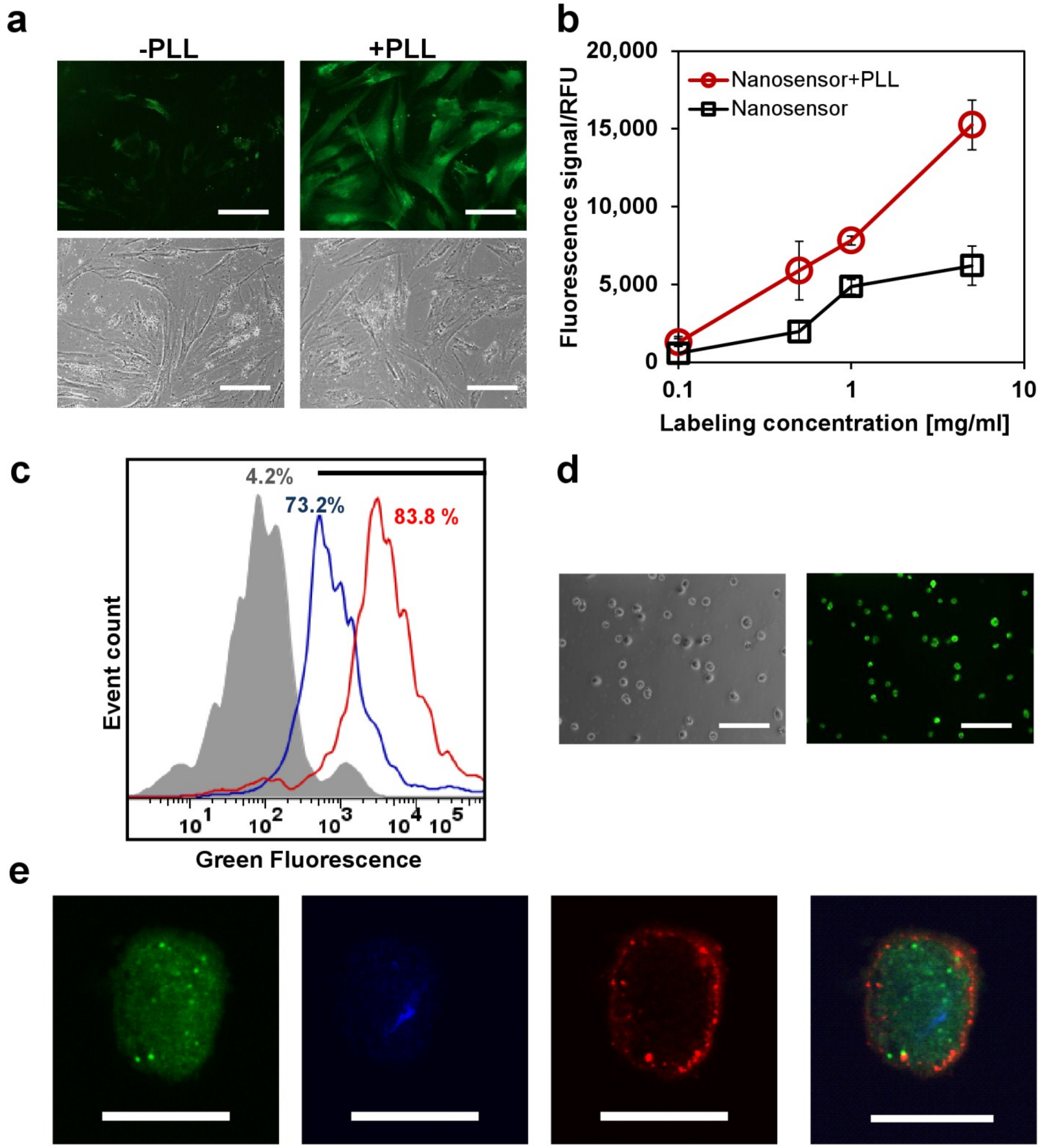

Supplementary Figure S2. Mesenchymal stem cell labeling with viability nanosensor. (**A**) Representative fluorescence and phase contrast images of MSCs labeled with PLL modified or unmodified nanosensors. (**B**) MSC fluorescence signal, measured as relative fluorescence units (RFU) after being labeled with PLL modified or unmodified nanosensors between the concentration range of 0.1 – 5 mg/ml. (**C**) Flow cytometry analysis of MSCs before (grey), after labeling with 0.3 mg/ml (blue) and 3

mg/ml (red) viability nanosensor. The positively gated population for each group is indicated above. (**D**) Fluorescence and phase contrast images of nanosensor labeled cells after trypsin dissociation. (**E**) Representative optical sections of a single MSC indicating: green (live nanosensor), blue fluorescence (nucleus), red (plasma membrane) and merged image (green + red + blue), Scale bar represents 20 μm. All values are mean ± SD, N≥3. Scale bars represent 100 μm unless otherwise stated.

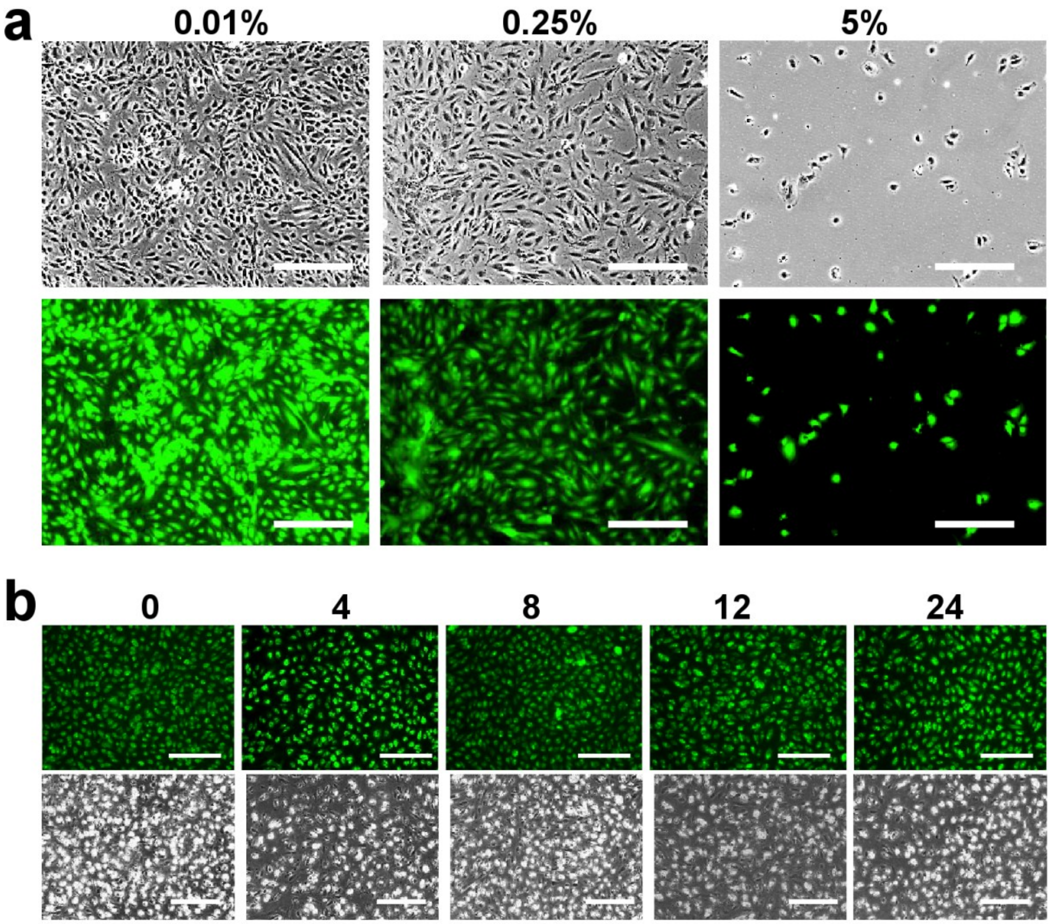

Supplementary Figure S3. Monitoring cell viability with nanosensors. (**A**) Representative phase contrast and fluorescence images of cells treated with dimethyl sulfoxide (DMSO: 0.01, 0.25 & 5% v/v) for 24 hours using the calcein acetomethoxy (CAM) assay. (**B**) Representative phase contrast and fluorescence images of nanosensor labeled cells treated with medium without DMSO for 0, 4, 8, 12 and 24 hours. Scale bar represents 100 μm.

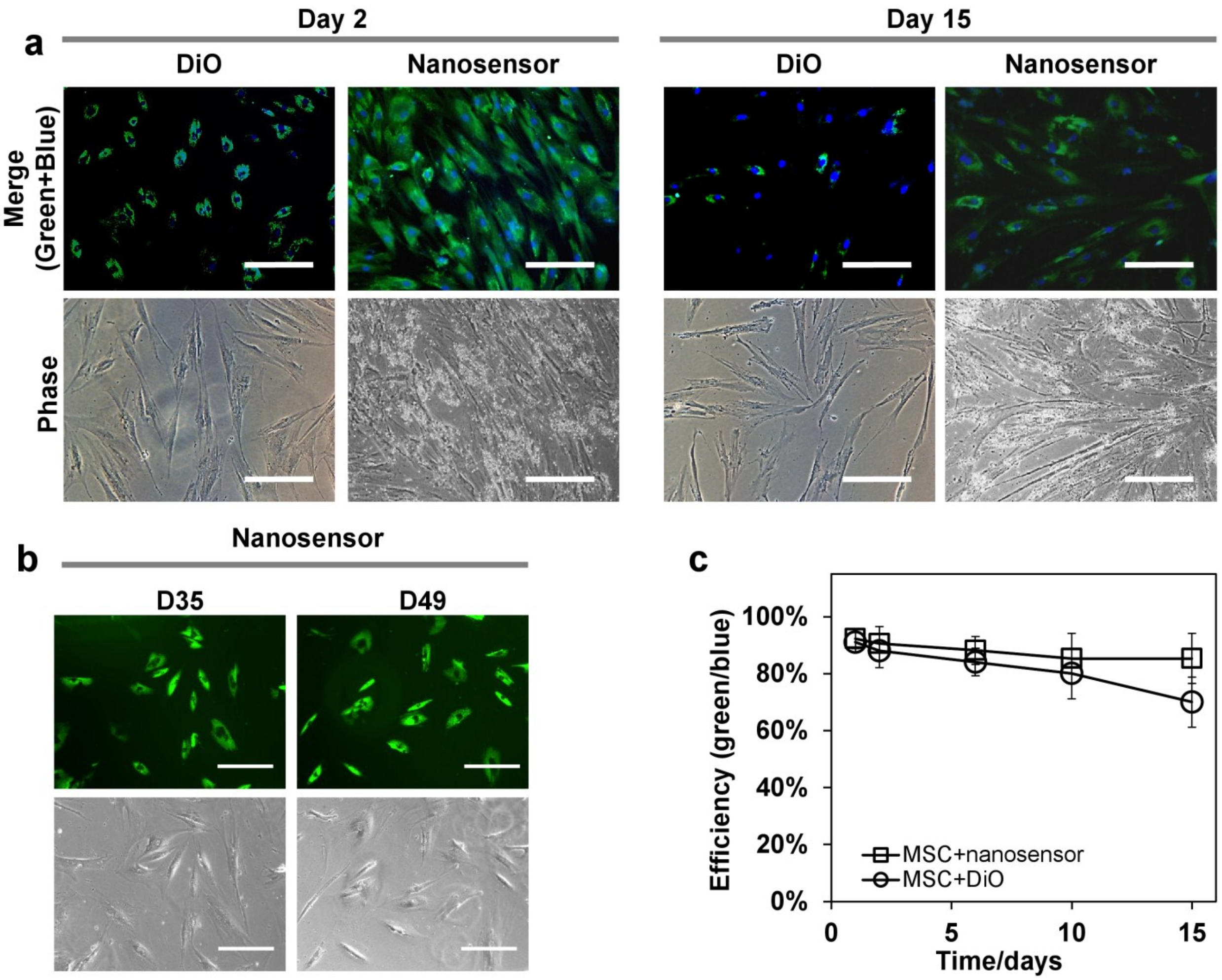

Supplementary Figure S4. Longitudinal cell tracking with DiO and nanosensors. (**A**) Representative merged fluorescence (green + blue) and phase contrast images of $DiIC_{18}(3)$ DiO and nanosensor labeled MSCs at day 1. Green from respective labeling groups (nanosensor & DiO), blue from Hoechst 33342 nuclei labeling stain. (**B**) Percentage of fluorescent cells at day 1 and 14 post labeling with DiO and nanosensors. (**C**) Green fluorescence & phase contrast images of nanosensor labeled MSCs at day 42, 49 and 56 post labeling. Values are mean ± SD, N=4. Scale bars represent 100 μm.

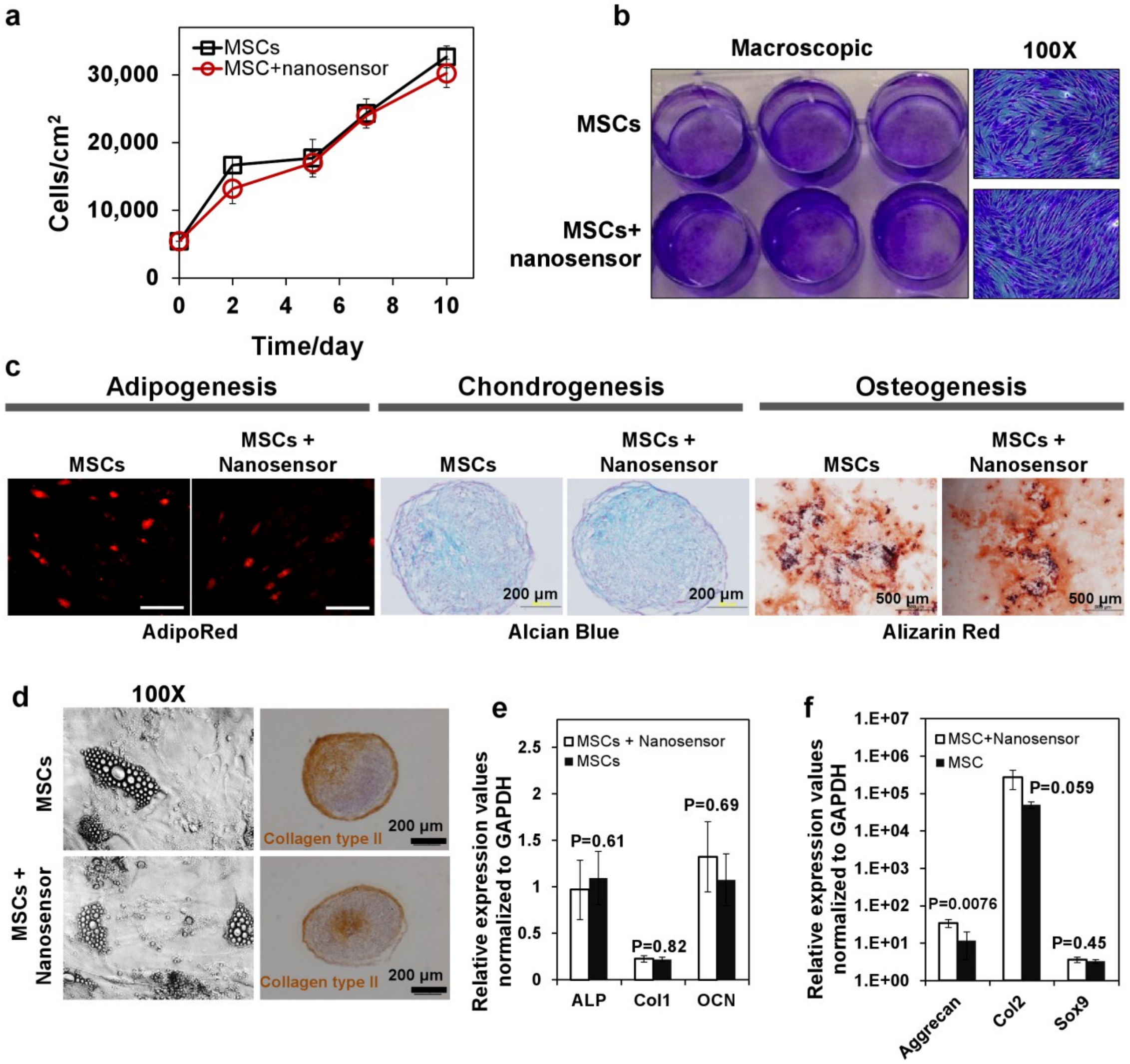

Supplementary Figure S5. Proliferation, 'stemness' and differentiation characteristics of nanosensor labeled MSCs. (**a**) Proliferation of nanosensor labeled MSCs and unmodified MSCs over 10 days. (**b**) Crystal violet stained colonies from colony forming unit-fibroblast (CFU-F) assay to evaluate the 'stemness' of unlabeled and nanosensor labeled MSCs. (**c**) Adipogenic, chondrogenic, and osteogenic differentiation of unlabeled and nanosensor labeled MSCs with AdipoRed, Alcian blue and Alizarin Red staining respectively. (**d**) Adipogenic lipid vacuoles and immuno-histochemistry for collagen type II of unlabeled and nanosensor labeled MSCs. Relative expression of the indicated gene of interest normalized to glyceraldehyde 3-phophate dehydrogenase (GAPDH) and its initial values (day 0) for (**e**) 'osteogenic' (alkaline phosphatase - ALP, collagen 1-Col1, osteonectin-OCN) and (**f**) 'chondrogenic' (aggrecan, Collagen 2-Col2 and Sox9) gene markers in both unlabeled and nanosensor labeled MSCs. Values are mean ± SD, N≥3. Scale bars represent 100 µm unless otherwise stated.

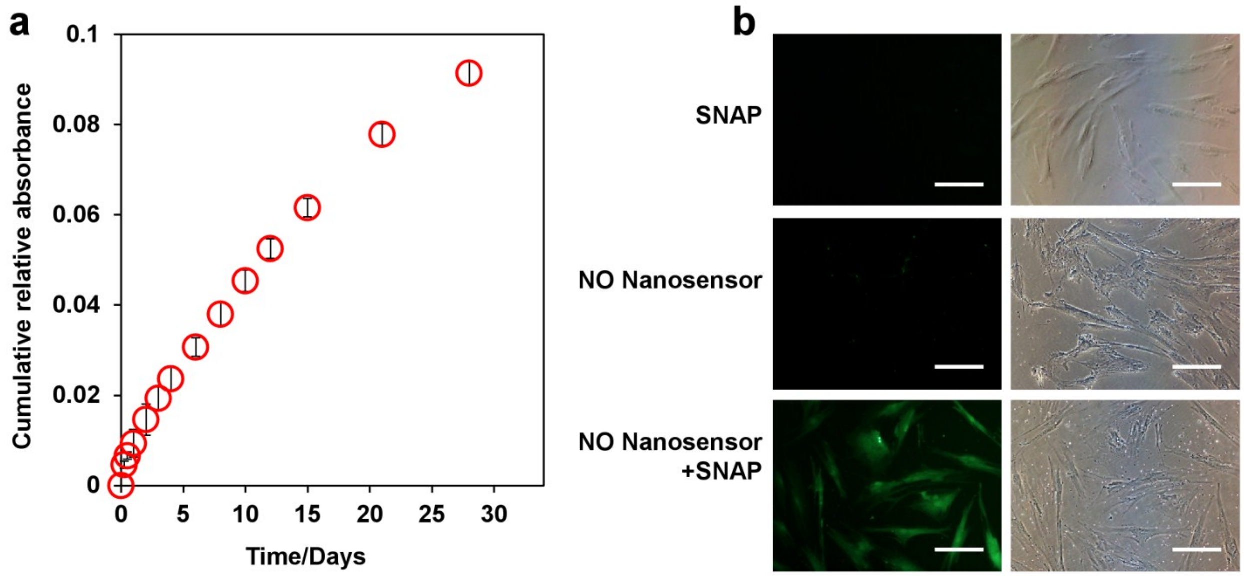

Supplementary Figure S6. Nitric oxide (NO) nanosensors. (**a**) Cumulative relative absorbance values (UV-VIS detection) of 4-Amino-5-Methylamino-2',7'-Difluorofluorescein diacetate (DAF-FM DA) released from NO nanosensors into the supernatant over a 28 day period. (**b**) Fluorescence and bright field images of MSCs treated with S-Nitroso-N-acetylpenicillamine (SNAP) alone, NO nanosensors alone, or the combination of SNAP and NO nanosensors. Values are mean ± SD, N≥3. Scale bars represent 100 μm.

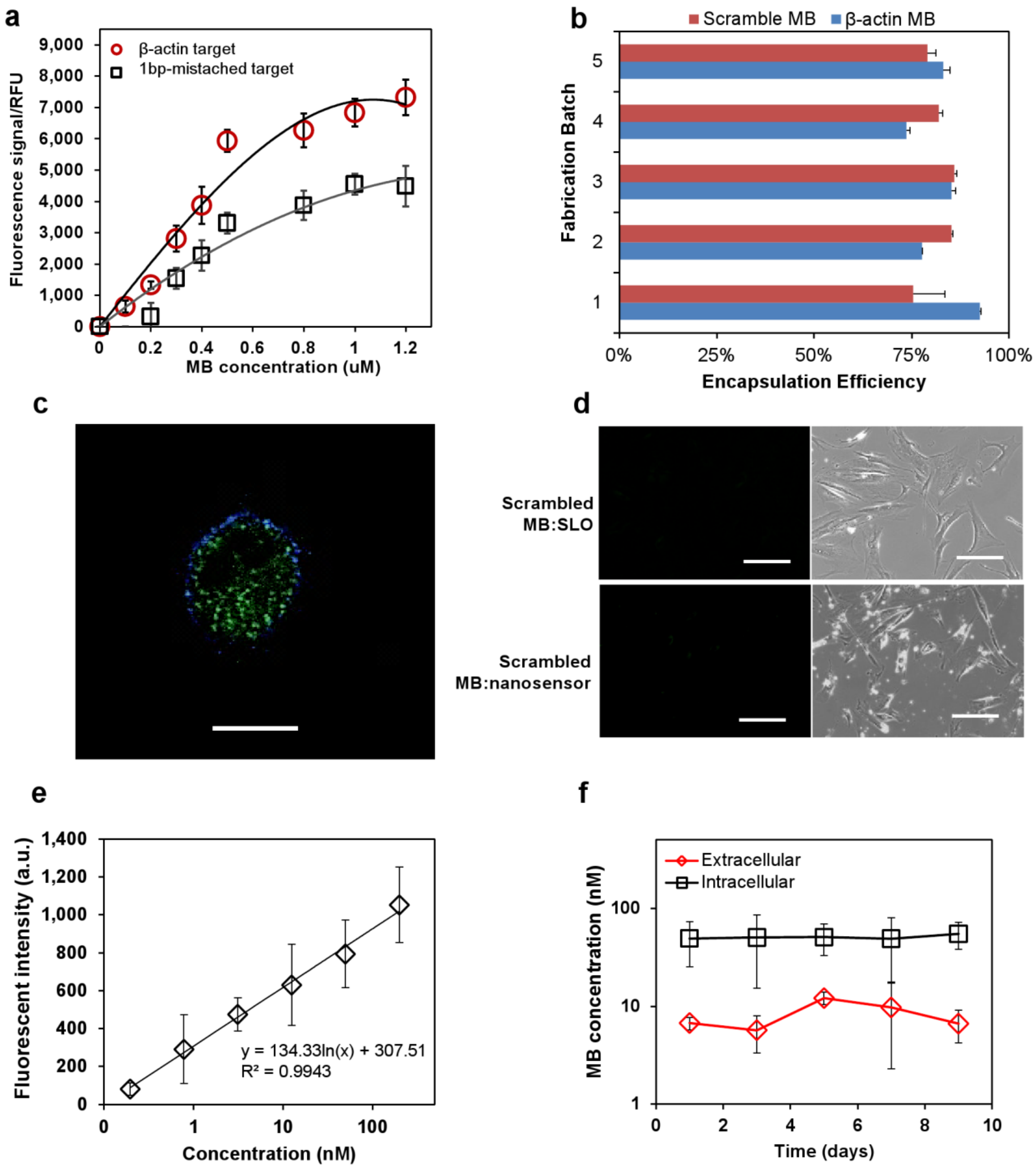

Supplementary Figure S7. Characterization of β-actin mRNA nanosensors. (**a**) Fluorescence intensity of solutions containing various amount of β-actin MBs incubated with either perfect target or 1 base-pair (bp) mismatched targets. (**b**) Encapsulation efficiency of Scrambled and β-actin MB-nanosensors from 5 independent batches (**c**) Intracellular distribution of SLO delivered MBs (green) within cell plasma membrane (blue) merged (main image). Scale bar: 20 μm. (**d**) Fluorescence and phase contrast images of mesenchymal stem cells (MSCs) labeled with scrambled sequence molecular beacons (MBs) delivered by streptolysin (SLO) or nanosensors (4 hrs). (**e**) Standard curve of fluorescence intensity from known Scrambled MB quantities treated with DNase I. (**f**) Intracellular and extracellular MB concentrations with respect to time. Values are mean ± SD, N=4. Scale bars: 100 μm.
